# Comparing the Quality of Cardiopulmonary Resuscitation Performed at the Over-the-Head Position and Lateral Position of Neonatal Manikin

**DOI:** 10.3389/fped.2019.00559

**Published:** 2020-01-20

**Authors:** Po-Yin Cheung, Hongmei Huang, Chenguang Xu, Jiang-Qin Liu, Joseph Y. Ting, Rosanna Wong, Winnie Lee, Yin Xue, Yanzhi Yi

**Affiliations:** ^1^Centre for the Studies of Asphyxia and Resuscitation and Departments of Pediatrics, Pharmacology and Surgery, University of Alberta, Edmonton, AB, Canada; ^2^Hong Kong University-Shenzhen Hospital, Shenzhen, China; ^3^Children's Medical Center, Chongqing YouYouBaoBei Women's and Children's Hospital, Chongqing, China; ^4^Shanghai First Maternity and Infant Hospital, Tongji University School of Medicine, Shanghai, China; ^5^British Columbia Women's Hospital, University of British Columbia, Vancouver, BC, Canada; ^6^Hong Kong Children's Hospital, Hong Kong, Hong Kong

**Keywords:** NRP®, simulation, CPR, quality performance, position

## Abstract

**Background:** Recent neonatal resuscitation guidelines suggest to perform chest compression (CC) at over-the-head (OTH) position instead of lateral position when further interventions including umbilical venous access are needed. Little information is available regarding the quality of cardiopulmonary resuscitation at different positions. Our study compared the quality of CC and ventilation at OTH position vs. lateral position in simulated neonatal resuscitation.

**Methods:** Thirty-nine neonatal practitioners who attended the NRP®-based Provider renewal course workshop participated this study. Laerdal QCPR infant model were used to collect the data (2-miutes continuous recording) on quality of CC and ventilation of all participants at OTH position and lateral position in randomized order, both coordinated with mask ventilation or endotracheal ventilation through a Neopuff^©^ T-piece system. The quality of CC and ventilation were compared. Participants also reported their demographics and opinions in anonymous questionnaires after the session.

**Results:** The quality of CC and ventilation was not different when CPR was performed at OTH position and lateral position, in both mask and endotracheal ventilation. When CPR was performed with endotracheal ventilation, there were small faster frequencies of CC and ventilation at OTH position, compared with those at lateral position (*p* = 0.004). Most participants (87%) liked the CC performed at OTH position and had no adverse feedback.

**Conclusions:** Performing CC at OTH position was generally well-received in simulated resuscitation; the quality of CC and ventilation at OTH position was not significantly different from that at lateral position, irrespective of mask or endotracheal ventilation.

## Introduction

**Figure 1:**
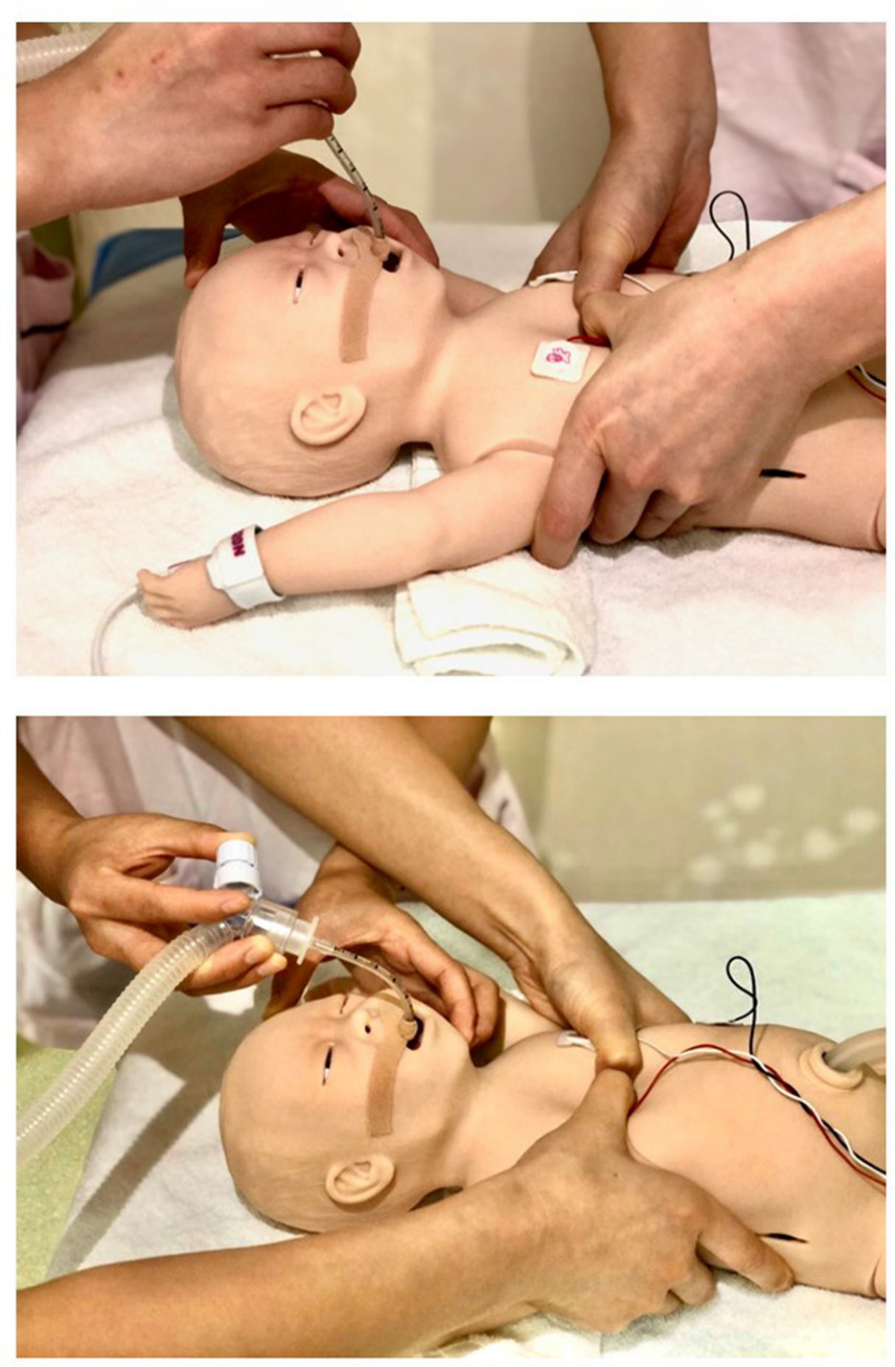
Cardiopulmonary resuscitation at lateral position **(upper panel)** and over-the-head position **(lower panel)**.

In the resuscitation of neonates at birth, interventions range from simple respiratory care of suction and provision of positive pressure ventilation through a bag and mask to cardiopulmonary resuscitation (CPR) with coordinated chest compressions (CC) and ventilation. Neonatal Resuscitation Program (NRP)® was started in late 80s and is designed to teach but not limit to practitioners and/or clinicians who attend the deliveries and resuscitate those at-risk neonates. NRP® guidelines recently recommended the performance of CC at the over-the-head (OTH) position instead of lateral position during CPR (1). The advantage of CC at OTH position is mainly related to make more space available and less interference for procedures including umbilical venous access and or umbilical venous catheter placement, when compared to the CC at lateral position (Figure 1). Either position however requires the coordination of two NRP® providers during CPR. Although the comparison in CPR quality between the two positions has been reported in adult resuscitation (2, 3), little information is available regarding the effect of NRP® providers in these two positions on the quality of CPR skills.

In this study, we aimed to compare the quality of CPR (ventilation and CC) of neonatal practitioners with CC performed at OTH position and lateral position. We hypothesized that the quality of ventilation and CC would be better when the CC was performed at the OTH position, compared to that with CC at the lateral position. Further, we studied the acceptance and user-friendliness of the two positions for CC.

## Methods

The study has received approval from the institutional ethics committee of the Hong Kong University Shenzhen Hospital.

**Figure 2:**
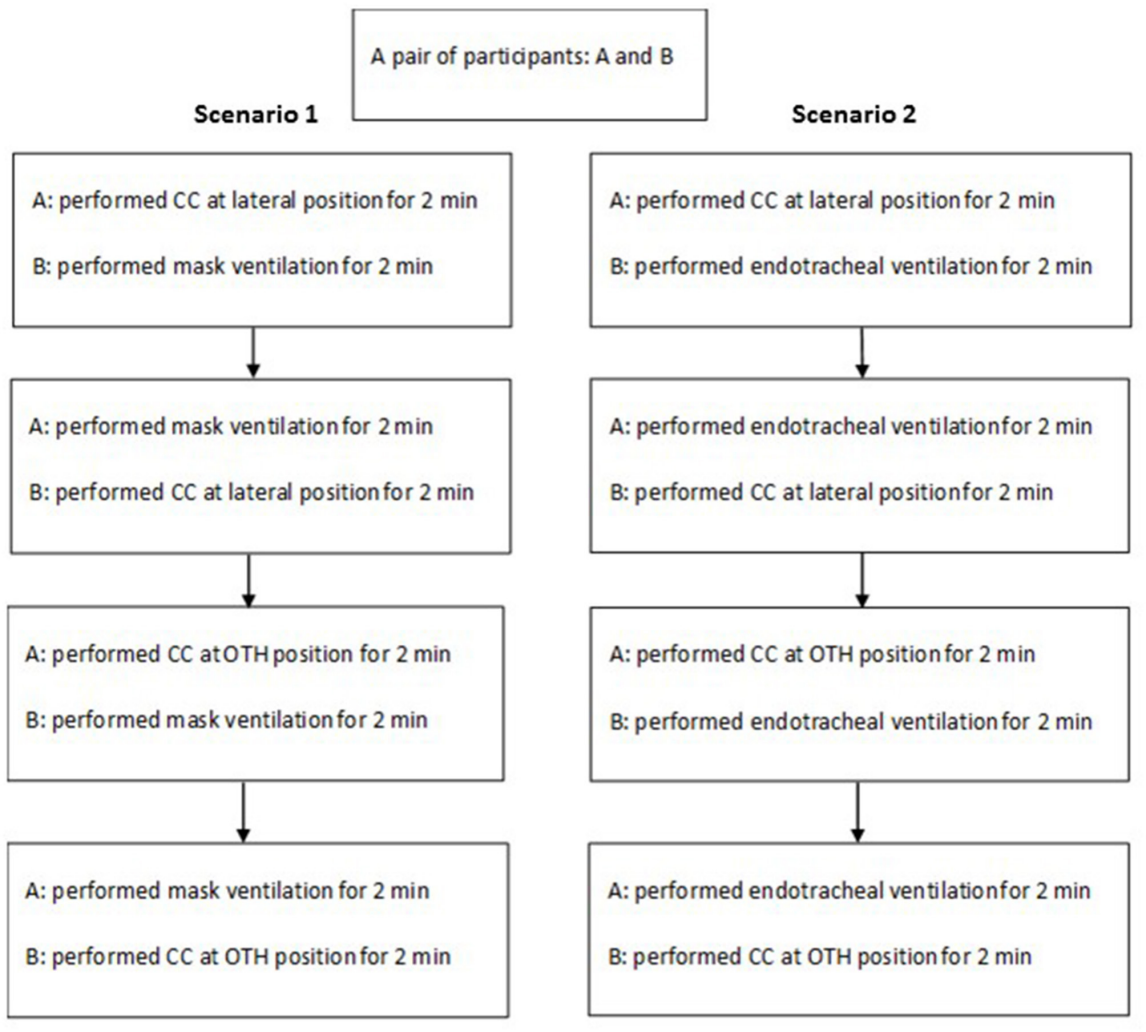
Study protocol.

In April 2017, 46 neonatal practitioners (doctors and nurses) attended the biannual NRP®-based Provider course workshop at the Hong Kong University Shenzhen Hospital. All the participants were approached, and informed consents were obtained. In the workshop, there were face-to-face performance skills stations on effective mask and endotracheal ventilation and coordinated CC during CPR as well as other components of NRP® curriculum. Integrated skills performance of each participant was first practiced and then evaluated using standardized simulated cases of asphyxiated newborns in the delivery room. The conduction of the integrated skills performance session was such that standardized simulated cases would have severe bradycardia of <60 bpm and led to the administration of CPR despite correct MR SOPA procedures. MR SOPA procedures include (in the following order): Mask adjustment, Reposition the head to ensure an open airway, Suction the mouth and nose, Open the mouth and lift the jaw forward, Pressure increase, and Alternate airway including endotracheal intubation. A pair of practitioners who participated in the session alternated their role in the provision of ventilation and CC in each case scenario in a random fashion. While the participants consented to the study, they did not have advanced knowledge of the case scenario. No prior pairwise practice was required in the evaluation. The CC was first performed at the lateral position and then at the OTH position after 2 min of CC (Figure 2). The quality of CPR was continuously recorded throughout the periods of CC at both lateral and OTH positions.

For the evaluation of the quality of CPR, Laerdal QCPR infant model and software (Laerdal Medical AS., Stavanger, Norway) were used. The performance of CPR was continuously recorded for 2 min. The quality of mask and endotracheal ventilation (rate and tidal volume) and of CC (rate, depth, chest rebound and position) was evaluated, as well as synchrony of ventilation and CC at 3:1 ratio. The demographics of participants were also collected including gender, occupation, the number of years of practice in NICU and experience of simulated training in neonatal resuscitation. Immediately following the completion of CPR simulation study, a brief survey regarding the preference of position and the respective reasons was conducted in all participants.

In this study, as per manufacturer's instructions of the QCPR program, we defined (a) accurate ventilation rate at 30 ± 20% (24–36) ventilation per min; (b) effective ventilation as >80% tidal volume at 20–40 ml; (c) accurate CC rate as an average CC rate of 90 ± 20% (72–108) compressions per min; (d) effective CC depth as >80% of CC depth reached 1/3 the anterior-posterior chest depth of manikin; (e) effective CC rebound as >80% of CC rebound were adequate; and (f) synchronized ventilation and CC as >80% CPR events (ventilation and compressions) with coordinated ventilation and CC at 3:1 ratio. Accurate position was considered as the lower one-third of the sternum. The quality of CPR of each participant, who had 2 min of CC at two positions during mask and endotracheal ventilation, was analyzed amounting a total of more than 35,000 CPR events.

Data are presented in mean ± SD and *n* (%) as appropriate. Data were compared using Student's *t*-test and Mann–Whitney *U*-test for parametric and non-parametric parameters, respectively. For the comparison of proportions, *z*-test was used. *P*-value of <0.05 was considered as significant. SigmaPlot v14.0 (Systat Software Inc., CA) was used for statistical analyses. Sample size was based on convenience (number of participants in the neonatal resuscitation workshop).

## Results

In the neonatal resuscitation workshop, there were 39 (85%) females, 28 (61%) doctors and 18 (39%) nurses. Among the 46 participants, 17 (37%) and 19 (41%) had 5–10 years and >10 years of NICU experience, respectively, and 30 (65%) had previous experience in simulated training in neonatal resuscitation. Thirty-nine (85% of 46) participants consented to the study and had complete data for analysis.

The quality of CC and ventilation was not different when CPR was performed at OTH position and lateral position, in both mask- and endotracheal- ventilation (Tables 1, 2). When CPR was performed with endotracheal ventilation, there were significantly but small faster frequencies of CC and ventilation at OTH position, compared with those at lateral position and that of CC happened during the final 30 s only (42 ± 6 vs. 39 ± 5; *p* = 0.004, respectively). Other parameters of CC were not different between the first and final quarter time (data not shown). Most participants (87%) liked the CC performed at OTH position and had no adverse feedback.

**Table 1:** Comparison of quality of chest compressions (CC) and ventilation when CC was performed at lateral position and over-the-head (OTH) position during cardiopulmonary resuscitation (CPR) with mask ventilation using T-piece [mean ± SD or *n* (%)].

**Quality of CPR**	**Lateral position**	**OTH position**	***P*-value**
**Quality of chest compressions**			
Frequency (events/min)	85 ± 10	85 ± 9	0.96
Accurate rate	35 (90%)	35 (90%)	1.00
Mean depth (mm)	39 ± 3	39 ± 3	0.73
Effective chest compressions depth	31 (80%)	31 (80%)	1.00
Effective chest compressions rebound	21 (54%)	22 (56%)	0.82
Accurate position	37 (95%)	38 (97%)	1.00
**Quality of ventilation**			
Frequency (events/min)	28 ± 3	28 ± 3	0.92
Accurate rate	37 (95%)	34 (87%)	0.23
Mean tidal volume (ml)	27 ± 8	25 ± 10	0.16
Effective ventilation	22 (56%)	21 (54%)	0.82
Synchrony between chest compressions and ventilation	39 (100%)	39 (100%)	1.00

**Table 2:** Comparison of quality of chest compressions (CC) and ventilation when CC was performed at lateral position vs. over-the-head (OTH) position during cardiopulmonary resuscitation (CPR) with endotracheal ventilation using T-piece [mean ± SD or *n* (%)].

**Quality of CPR**	**Lateral position**	**OTH position**	***P*-value**
**Quality of chest compressions**			
Frequency (events/min)	83 ± 10	88 ± 11	**0.004**
Accurate rate	34 (87%)	35 (90%)	0.73
Mean depth (mm)	39 ± 4	39 ± 3	0.69
Effective chest compressions depth	31 (80%)	34 (87%)	0.36
Effective chest compressions rebound	18 (46%)	22 (56%)	0.37
Accurate position	38 (97%)	39 (100%)	1.00
**Quality of ventilation**			
Frequency (events/min)	28 ± 3	30 ± 4	**0.004**
Accurate rate	36 (92%)	37 (95%)	0.65
Mean tidal volume (ml)	23 ± 10	20 ± 11	0.09
Effective ventilation	20 (51%)	16 (41%)	0.36
Synchrony between chest compressions and ventilation	39 (100%)	39 (100%)	1.00

*Bold values are statistically significant*.

In the post-study survey, 87% (*n* = 34) of participants preferred the performance of CC at OTH position with the remaining (13%, *n* = 5) favoring CC at lateral position. A qualitative review of the survey (by HH and CX) found ease of performance and degree of exhaustion as reasons for preference of CC at the OTH position over lateral position. The main reason for favoring CC at the lateral position was related to the habit of performance of CPR.

## Discussion

Although most newborn infants will have a smooth transition from fetal to neonatal life, it is estimated that approximately 0.1% neonates of term gestation will require CPR with coordinated CC and ventilation, and a higher incidence is found in preterm neonates. When CC is needed during CPR, in the 2015 guidelines, NRP® recommends the compressor to move to the head of the bed while the person providing ventilation moves to the side (1). The reason for this recommendation is related to the fact of a high probability of an emergency vascular access for CPR. The latter requires an umbilical venous access or catheter placement. Thus, the OTH position of CC allows more space for procedures at the umbilicus.

Indeed, the performance of CC at the lateral position may allow more space in the airway interventions at the head and neck region to ensure effective ventilation including mask adjustment, airway opening, and endotracheal intubation. Mask ventilation is usually performed by the practitioner at OTH position. When endotracheal tube has not been established, CC at OTH may interfere mask ventilation or mask readjustment. However, in our observational study, the proportions of effective mask ventilation using T-piece were similar at the two positions (56% at lateral position vs. 54% at OTH position). Further, in the latest NRP® guidelines, it is recommended to intubate to ensure effective ventilation prior to the administration of CC.

Maisch et al. reported better CPR quality (better ventilation with correct tidal volume, more CC and less hand-off time) by a single rescuer but similar CPR quality by two rescuers, when CPR was performed at the OTH compared with at the lateral position (2, 3). Interestingly, Chi et al. did not find significant differences between the kinematics, compression forces, depths, and frequencies obtained in both CPR positions as practiced by experienced providers (4). We however did not identify any similar study in neonatal resuscitation which is different from that in adults in respect to size and thus space availability in CPR, in addition to the clinical scenario.

We found the quality of CC and ventilation was not different between the two positions. This is reassuring and therefore CC at the OTH position should be favored for the logistic reasons stated. Interestingly, when CC were performed at the OTH position during endotracheal ventilation which is the standard of care in neonatal CPR as stated in the 2015 NRP® guidelines, we found a significant but modest increase in the CC and ventilation rates (88 vs. 83 compressions/min of CC and 30 vs. 28 breaths/min of ventilation at OTH position and lateral position, respectively). There are reports showing better basic life support with CPR at OTH position by a single rescuer with higher number of CC, tidal volume and percentage of correct tidal volume delivered (2, 5). Maisch et al. reported a higher number of CC during a 2-min test of simulated adult CPR at OTH position, compared to lateral position, in a single rescuer CPR (2). Although they showed similar numbers of inflation, there was higher tidal volume and percentage of correct tidal volume delivered in the group of OTH position than those at lateral position. However, Maisch et al. reported that in a two-professional-rescuer CPR scenario, standard CPR enables a quantitatively better resuscitation than OTH CPR (3). While the adult CPR comparison was conducted in the settings of a single rescuer, the advantages of OTH position are related to practicability, stationary rescuer's position, and confined space available for CPR. These factors may not be applicable in neonatal CPR and the cause(s) is yet to be determined. Nevertheless, the post-test survey reported an overwhelmingly preference and thus the practicability of OTH position (87 vs. 13% for lateral position).

## Limitations

There are several limitations in our study. First, this is an observational study in neonatal manikin. The result may not be applicable to clinical practice. Second, the sample size is small and precludes from detecting the difference in quality of ventilation and CC between the two positions including differences in the unexperienced and experienced sub-groups. The starting of CC at the lateral position and followed by the OTH might have favored the better quality of CPR at the OTH. However, performing CC at the lateral position is the conventional practice and this was usually provided first in the clinical settings. Therefore, we believe that the differences in quality of CPR between the two positions remain modest. Nevertheless, further clinical study is required to confirm our findings.

Based on our preliminary study, we found that performing CC at OTH position was generally well-received in simulated resuscitation. The quality of CC and ventilation at OTH position was not significantly different from that at lateral position, irrespective of mask or endotracheal ventilation.

## Data Availability Statement

The raw data supporting the conclusions of this manuscript will be made available upon request to the corresponding author, without undue reservation to any qualified researcher.

## Ethics Statement

The study has received approval from the institutional ethics committee of the Hong Kong University Shenzhen Hospital. The participants provided their written informed consent to participate in this study.

## Author Contributions

P-YC, HH, and CX: substantial contributions to the conception. HH, CX, J-QL, JT, RW, WL, YX, and YY: acquisition. P-YC: drafting the work. All authors: design of the work, analysis or interpretation of data for the work, revising it critically for important intellectual content, provide approval for publication of the content, and agree to be accountable for all aspects of the work in ensuring that questions related to the accuracy or integrity of any part of the work are appropriately investigated and resolved.

### Conflict of Interest

The authors declare that the research was conducted in the absence of any commercial or financial relationships that could be construed as a potential conflict of interest.
